# Usando Troponina de Alta Sensibilidade para Melhorar a Previsão de Risco após Cirurgia Não Cardíaca – Já Chegamos Lá?

**DOI:** 10.36660/abc.20240140

**Published:** 2024-06-03

**Authors:** António Tralhão

**Affiliations:** 1 Unidade Local de Saúde de Lisboa Ocidental Unidade de Cuidados Intensivos Cardíacos Serviço de Cardiologia Lisboa Portugal Unidade de Cuidados Intensivos Cardíacos – Serviço de Cardiologia – Unidade Local de Saúde de Lisboa Ocidental, Lisboa – Portugal; 2 Nova Medical School Lisboa Portugal Nova Medical School, Lisboa – Portugal

**Keywords:** Injúria Miocárdica, Cirurgia Não Cardíaca, Troponina Ultrasensível, Classificação de Risco

A perturbação homeostática relacionada à cirurgia não cardíaca é um gatilho bem descrito para morbidade e mortalidade cardiovascular no pós-operatório imediato e tardio.^1,2^ Por meio de múltiplos mecanismos, incluindo inflamação e hipercoagulabilidade, instabilidade hemodinâmica e perda sanguínea, hipoxemia ou suspensão de terapias protetoras, condições cardíacas crônicas conhecidas ou silenciosas, como doença arterial coronariana, disfunção ventricular esquerda ou doença valvar, podem servir como substrato para isquemia e infarto do miocárdio, insuficiência cardíaca ou arritmias.^2^

Epidemiologicamente, uma taxa significativa de complicações cardíacas, combinada com um imenso número de pacientes submetidos a procedimentos cirúrgicos não cardíacos por ano, cria um grande desafio à saúde pública, mudando necessariamente o foco para a prevenção.^3^ Tradicionalmente, a avaliação de risco tem sido baseada em fatores do paciente, tipo de cirurgia e urgência da intervenção, que estão incluídos, embora com pesos diferentes, nos escores de risco disponíveis.^4,5^ Infelizmente, a capacidade discriminativa das pontuações existentes está longe de ser ideal, deixando espaço para ferramentas complementares como os biomarcadores. Especificamente, foi demonstrado que a liberação de troponina cardíaca (cTn) na corrente sanguínea, refletindo lesão multifatorial de cardiomiócitos, sinaliza aumento de morbidade e mortalidade quando elevada no período perioperatório.^6^ No entanto, e até agora, a integração deste chamado MINS ("lesão miocárdica após cirurgia não cardíaca") com a mais familiar avaliação de risco clínico, na tarefa de previsão de risco continua a ser uma questão a ser devidamente respondida.

Nesta edição da revista, Gomes et al.,^7^ apresentam uma análise retrospectiva unicêntrica de 2.230 pacientes internados na Unidade de Terapia Intensiva após cirurgia não cardíaca por um periodo de 5 anos, acompanhados por uma mediana de 6,7 anos.^7^ Eles descobriram que o MINS era bastante frequente, pois ocorria em quase um em cada 10 pacientes, um número que não foge dos estudos publicados anteriormente.^8,9^ Tal como biologicamente esperado, o risco clínico correlacionou-se com a probabilidade de MINS, anunciando uma maior carga de doenças cardiovasculares.

O MINS foi então combinado com a avaliação de risco com base em três métricas (risco CV, RCRI e risco cirúrgico), criando quatro subgrupos de pacientes ao emparelhar o risco (+/-) com o MINS (+/-), para cada uma dessas métricas. A grande maioria dos pacientes tinha baixo risco e nenhum MINS e serviu como população de referência. A categorização de alto risco foi substancialmente diferente de acordo com risco CV (26,1%), RCRI (0,9%) e risco cirúrgico (14,6%). A análise de sobrevivência mostrou que os pacientes que apresentavam maior risco e MINS tinham pior prognóstico, mas também que os pacientes MINS com baixo risco na verdade se saíram pior do que os pacientes considerados de alto risco, mas nos quais MINS não foi detectado, destacando uma sensibilidade reduzida da avaliação clínica para detectar aumento mortalidade em pacientes de baixo risco. Uma exceção a esse padrão foi observada para pacientes com RCRI elevado, nos quais o MINS não separou a probabilidade de sobrevivência, possivelmente relacionado ao pequeno número de pacientes nesse grupo. Depois de contabilizar a gravidade geral do paciente através da pontuação SAPS 3, o MINS manteve o impacto prognóstico independente. Estes resultados foram ainda reforçados pelo recurso a três metodologias estatísticas diferentes, mostrando a melhoria da capacidade discriminativa da cTn quando adicionada à estratificação de risco clínico. Dados estes resultados, o papel da medição da cTn na avaliação do risco cardiovascular após cirurgia não cardíaca parece merecer uma reavaliação cuidadosa.

As diretrizes atuais do ESC sobre cirurgia não cardíaca recomendam a medição de cTnI ou cTnT antes da cirurgia e 24 e 48 horas depois, mas apenas em pacientes com alta.^4^ As diretrizes da ACC/AHA sobre avaliação cardiovascular perioperatória e manejo de pacientes submetidos à cirurgia não cardíaca restringem o papel da cTn a pacientes que apresentam sinais de isquemia miocárdica, levantando preocupações sobre a falta de estratégias de manejo após MINS. Mais recentemente, uma diretriz focada no papel da cTn na avaliação do risco cirúrgico não cardíaco sugere a determinação pré-operatória e pós-operatória da cTn para melhorar a previsão de risco, com um nível moderado de evidência.^10^

Antes que a medição da cTn possa ser recomendada a todos os pacientes submetidos à cirurgia não cardíaca, diversas questões não resolvidas, algumas delas levantadas por Gomes et al.,^7^ permanecem a serem abordadas em estudos futuros. Em primeiro lugar, a área sob a curva ROC para predição de mortalidade foi modesta mesmo após a adição de cTn ao modelo. Em segundo lugar, como a elevação da cTn é multifatorial e está associada a complicações não cardiovasculares, seria relevante diferenciar a mortalidade cardíaca da não cardíaca, pois isso poderia elucidar mecanismos fisiopatológicos e possivelmente implicar diferentes estratégias de manejo. Em terceiro lugar, o desempenho de outros escores de risco, como a Calculadora de Risco Cirúrgico ou a Ferramenta de Risco de Resultado Cirúrgico, que possuem melhor poder discriminativo, não foi testado.

Finalmente, a questão mais difícil, clinicamente mais importante, e ainda sem resposta, diz respeito à ausência de uma via de diagnóstico e tratamento rentável que, através de terapias modificadoras da doença, resultasse em melhores resultados para os pacientes, evitando ao mesmo tempo testes a jusante desnecessários e intervenções invasivas. Embora o trabalho de Gomes et al.,^7^ represente mais um passo na adaptação da previsão de risco após cirurgia não cardíaca, a aplicabilidade de uma estratégia de tratamento melhorada com cTn continua necessitando de uma análise cuidada em estudos clínicos bem concebidos.
