# Supplementary material for: Usando Troponina de Alta Sensibilidade para Melhorar a Previsão de Risco após Cirurgia Não Cardíaca – Já Chegamos Lá?
Source: Arq Bras Cardiol. 2024 Jun 3;121(4):e20240140. [Article in Portuguese] doi: 10.36660/abc.20240140 (PMC11164440; doi:10.36660/abc.20240140)
Supplement: Translation

# Translation: Using High-Sensitivity Troponin for Enhancing Risk Prediction after Non-Cardiac Surgery – Are We There Yet?

**DOI:** 10.36660/abc.20240140i

**Authors:** António Tralhão

**Affiliations:** 1 Lisboa Portugal Unidade de Cuidados Intensivos Cardíacos – Serviço de Cardiologia – Unidade Local de Saúde de Lisboa Ocidental, Lisboa – Portugal; 2 Lisboa Portugal Nova Medical School, Lisboa – Portugal

**Keywords:** Myocardial Injury, Non-Cardiac Surgery, High-Sensitivity Troponin, Risk Classification

The homeostatic disruption related to non-cardiac surgery is a well-described trigger for cardiovascular morbidity and mortality in the early and late post-operative periods.^1,2^ Through multiple mechanisms, including inflammation and hypercoagulability, hemodynamic instability and blood loss, hypoxemia or suspension of protective therapies, known or silent chronic cardiac conditions like coronary artery disease, left ventricular dysfunction or valve disease may serve as the substrate for myocardial ischemia and infarction, heart failure or arrhythmias.^2^

Epidemiologically, a significant rate of cardiac complications, combined with an immense number of patients undergoing non-cardiac surgical procedures *per annum* creates a major public healthcare challenge, necessarily shifting the focus towards prevention.^3^ Traditionally, risk assessment has been based on patient factors, type of surgery, and urgency of the intervention, which are included, albeit with different weighing, in the available risk scores.^4,5^ Unfortunately, the discriminative ability of existing scores is far from optimal, leaving room for complementary tools like biomarkers. Specifically, the bloodstream release of cardiac troponin (cTn), reflecting multifactorial cardiomyocyte injury, has been shown to signal increased morbidity and mortality when elevated in the perioperative period.^6^ However, and until now, the integration of this so-called MINS ("myocardial injury after non-cardiac surgery") with the more familiar clinical risk evaluation in the task of risk prediction remains a question to be properly answered.

In this issue of the Journal, Gomes et al.^7^ present a single-center retrospective analysis of 2230 patients admitted to the Intensive Care Unit after non-cardiac surgery for a 5 years-period, followed by a median of 6.7 years.^7^ They found that MINS was quite frequent, as it occurred in almost one out of 10 patients, a number that does not deviate from previously published studies.^8,9^ As biologically expected, clinical risk correlated with the likelihood of MINS, heralding a greater cardiovascular disease burden.

MINS was then combined with risk assessment based on 3 metrics (CV risk, RCRI, and surgical risk), creating four patient subgroups by pairing risk (+/-) with MINS (+/-), for each of these metrics. The large majority of patients had both low-risk and no MINS and served as the reference population. High-risk categorization was substantially different according to CV risk (26.1%), RCRI (0.9%), and surgical risk (14.6%). Survival analysis showed that patients displaying both higher risk and MINS had the worse prognosis but also that MINS patients with low-risk actually fared worse than patients deemed at high risk but in whom MINS was not detected, highlighting a reduced sensitivity of clinical assessment to detect increased mortality in low-risk patients. An exception to this pattern was noted for patients with an elevated RCRI, in which MINS did not separate survival probability, possibly relating to the small number of patients in this group. After accounting for overall patient severity through SAPS 3 scoring, MINS maintained independent prognostic impact. These findings were further strengthened by resorting to three different statistical methodologies, showing the improved discriminative ability of cTn when added to clinical risk stratification. Given these results, the role of cTn measurement in the assessment of cardiovascular risk after non-cardiac surgery seems to merit a thoughtful reappraisal.

Current ESC Guidelines on non-cardiac surgery recommend either cTnI or cTnT measurement before surgery and at 24 and 48 hours afterward, but only in high-patients.^4^ ACC/AHA guidelines on perioperative cardiovascular evaluation and management of patients undergoing non-cardiac surgery restrict the role of cTn to patients showing signs of myocardial ischemia, raising concerns about the lack of management strategies after MINS. More recently, a focused guideline on the role of cTn in non-cardiac surgical risk assessment suggests both preoperative and postoperative cTn determination to enhance risk prediction, with a moderate level of evidence.^10^

Before cTn measurement can be recommended to all patients undergoing non-cardiac surgery, several unresolved questions, some of them raised by Gomes et al.^7^ remain to be addressed in future studies. Firstly, the area under the ROC curve for mortality prediction was modest even after the addition of cTn to the model. Secondly, as cTn elevation is multifactorial and associated with non-cardiovascular complications, it would be relevant to differentiate cardiac from non-cardiac mortality, as this might elucidate pathophysiological mechanisms and possibly imply different management strategies. Thirdly, the performance of other risk scores such as the Surgical Risk Calculator or the Surgical Outcome Risk Tool, which have better discriminative power, was not tested.

Finally, the most difficult, clinically most important, and yet unanswered question pertains to the absence of a cost-effective diagnostic and treatment pathway that, through disease-modifying therapies, would result in better patient outcomes whilst avoiding unnecessary downstream tests and invasive interventions. Although the work by Gomes et al.^7^ represents one more step in tailoring risk prediction after non-cardiac surgery, the applicability of a cTn-enhanced management strategy remains to be carefully scrutinized in well-designed clinical studies.
